# Estimating cardiovascular disease incidence from prevalence: a spreadsheet based model

**DOI:** 10.1186/s12874-016-0288-y

**Published:** 2017-01-23

**Authors:** Xue Feng Hu, Kue Young, Hing Man Chan

**Affiliations:** 1 Department of Biology, University of Ottawa, 180B, Gendron Hall, 30 Marie Curie, Ottawa, ON K1N 6 N5 Canada; 2 School of Public Health, University of Alberta, 3-387, Edmonton Clinic Health Academy, 11405-87 Avenue, Edmonton, AB T6G 1C9 Canada

**Keywords:** Incidence, Prevalence, Model, Cardiovascular disease, NHANES, Canadian Community, Health Survey

## Abstract

**Background:**

Disease incidence and prevalence are both core indicators of population health. Incidence is generally not as readily accessible as prevalence. Cohort studies and electronic health record systems are two major way to estimate disease incidence. The former is time-consuming and expensive; the latter is not available in most developing countries. Alternatively, mathematical models could be used to estimate disease incidence from prevalence.

**Methods:**

We proposed and validated a method to estimate the age-standardized incidence of cardiovascular disease (CVD), with prevalence data from successive surveys and mortality data from empirical studies. Hallett’s method designed for estimating HIV infections in Africa was modified to estimate the incidence of myocardial infarction (MI) in the U.S. population and incidence of heart disease in the Canadian population.

**Results:**

Model-derived estimates were in close agreement with observed incidence from cohort studies and population surveillance systems. This method correctly captured the trend in incidence given sufficient waves of cross-sectional surveys. The estimated MI declining rate in the U.S. population was in accordance with the literature. This method was superior to closed cohort, in terms of the estimating trend of population cardiovascular disease incidence.

**Conclusion:**

It is possible to estimate CVD incidence accurately at the population level from cross-sectional prevalence data. This method has the potential to be used for age- and sex- specific incidence estimates, or to be expanded to other chronic conditions.

## Background

Disease incidence and prevalence are core indicators of population health. Accurate estimates of chronic diseases prevalence and incidence are essential for assessing population burden of disease and determining health priorities [1]. Incidence (rate of new cases among those at risk) is a better indicator of the progress of a disease epidemic, in comparison to prevalence (fraction of the population with the condition) for several reasons. First, change in prevalence lags behind the actual changes in population risk and incidence. Second, prevalence reflects historical trend and accumulation of cases, rather than recent incidence change. Many factors other than recent incidence change, such as population aging and survival improvement may influence the change in prevalence [2]. This is especially relevant to conditions such as myocardial infarction (MI) or stroke, a considerable portion of which are silent or transitory [3, 4]. Some affected individuals may not seek medical care, although they are at similar risk for adverse outcomes such as mortality when compared to individuals with detectable signs and symptoms. Many of the first occurrences of MI and stroke are fatal [5], such that recorded incidence of such conditions typically based on hospitalized cases is likely to underestimate the actual disease burden.

The most direct approach to estimating cardiovascular disease incidence is through longitudinal observational studies. Such studies are time-consuming, expensive, and are disease specific. Electronic health record systems and disease registry systems provide new sources of incidence estimates, but these are not available in most of the developing countries. Alternatively, incidence could be estimated by mathematical models, with prevalence data at multiple time points, population demographic change, and mortality as fundamental input. Prevalence of cardiovascular disease, typically from cross-sectional surveys, are generally more easily obtainable [6]. Mathematical models with varying degrees of sophistication have been developed, including Hallett’s method to estimate HIV incidence in Africa [7–9]. Theoretically, Hallett’s framework should be applicable in different settings and for other diseases as long as the disease is not reversible and local population/mortality data are available [6, 9]. However, the assumptions in this model were based on the HIV epidemic in Africa, and may not be applicable for cardiovascular diseases in a developed country.

Developing and testing a prevalence-incidence model (PI model) for cardiovascular diseases has both global and local significance. In Canada, for example, it is no longer possible to estimate the incidence of self-reported cardiovascular disease after the closing of the National Population Health Survey (NPHS) in 2012 [10]. While electronic health record systems are being set up in many locations across Canada, there is as yet no linked, national database. Reporting of all heart attack episodes (including silent cases and fatal cases) were rare, although hospitalized heart attack incidence rate was more frequently reported [11]. The PI model would be especially relevant for remote areas, in which cohort studies or electronic health record systems are likely to be unavailable. A PI model would also help to identify the gap between reported and true cardiovascular disease incidence, indicating priorities for health services planning.

In this study, we propose to modify the Hallett’s method and test its applicability to estimating cardiovascular disease incidence in the North America setting. We shall determine the hazard/probability of developing new cases across the time interval between two cross-sectional surveys and generate age-standardized incidence rates. We shall compare model estimated incidence rates with those obtained from cohort studies and population monitoring programs. If sufficient waves of cross-sectional surveys are available, the model could potentially also detect trends.

## Methods

### The HIV prevalence-incidence model

Among PI models with different complexities, the one proposed by Hallett et al. has been widely cited and used [9]. Detail of model derivation and validation has been published elsewhere [9]. Briefly, the difference between observed prevalence in the second survey and the expected prevalence (estimated from prevalence in the first survey and survival fraction based on mortality) provides the incident cases; the proportion of disease-negative people, the mortality rate for these people and the time interval between the two cross-sectional surveys together generate the number of person-years. Age- and gender- specific prevalence and mortality should be used to obtain more accurate estimates. Mortality of people with/without the health outcome of interest can be based on population vital statistics or the literature. The model assumes prevalence and mortality are constant during the interval of two successive surveys to keep the model simple and easy to use.

### Model adjustment and validation for cardiovascular diseases

Hallett’s method should work for other diseases and settings as long as the disease is not reversible. We made two major modifications to Hallett’s method to better fit cardiovascular diseases. First, the survival fraction of disease positive patients is estimated as a function of 30-day case fatality, 1-year, 5-year and 10-year survival/mortality rate whenever possible, instead of assuming a constant mortality rate between the time intervals of two surveys. Second, we calculated the age-standardized incidence rate to make the estimate more comparable to other health statistics in its target population.

### Model validation

We tested the performance of our modified Hallett’s method in two steps. First, we applied our model to estimate myocardial infarction (MI) incidence in the US population and compared estimated values to reported incidence rates from the national environmental public health tracking network (Tracking Network) of U.S. Centers for Disease Control and Prevention (CDC), existing epidemiological studies and population statistics. We chose MI in the US population as this condition was one of the most well studied cardiovascular outcomes. The mortality, prevalence, and survival data were relatively accurate and robust. Second, we expanded the outcome to the broader category of any heart disease (HD), which also includes heart failure and other forms of heart diseases, and compared incidence estimates with observed incidences from a national representative cohort study, in Canada. The cross-sectional surveys and the longitudinal survey we used share the same sampling framework and chronic disease module in their questionnaires. In the text hereafter, heart disease refers to combined MI, angina and heart failure in the Canadian population without further specification.

### Data source

For the United States, we chose the National Health and Nutrition Examination Survey (NHANES), which provides MI prevalence estimates in the US population at two-year intervals from 1999 to 2012 [12]. NHANES is a repeated cross-sectional survey of a nationally representative sample of the US population, with a multistage, stratified sampling design. Our study population included all participants aged 35 years and older in the 7 consecutive waves of the survey. Participants who answered “Yes” to questionnaire item MCQ160e (“Has a doctor or other health professional ever told you that you had heart attack (also called myocardial infarction)”) will be classified as prevalent MI cases in this study. Sex-specific mortality data for MI patients were calculated from hospitalized mortality and 2-year mortality after hospital discharge [13, 14]. Mortality data for the MI-negative population were calculated as the difference between all-cause mortality rate and the mortality rate of MI, from “Underlying Cause of Death 1999-2011” on the CDC WONDER Online Database [15]. Hospitalized MI incidence data were based on 26 States which participated in the CDC environmental public health tracking program [16].

For Canada, we selected the Canadian Community Health Survey (CCHS), which provides prevalence of self-reported heart diseases in the Canadian population, also at two-year intervals from 2001 to 2011 [17]. The CCHS is a series of cross-sectional surveys conducted biannually before 2007 and yearly since then. It uses a stratified, multistage probability sampling design. It collects information on health status, health care utilization and health determinants for the Canadian population. In this study, we identified participants aged 12 years and older in 6 consecutive waves of the survey. Participants who answered “Yes” to questionnaire item CCQ121 (“Have you had heart diseases which lasted 6 months or more and have been diagnosed by a health professional?”) were classified as prevalent heart disease cases. The mortality rate of heart disease was calculated as the total of mortality from MI, angina and heart failure. Mortality rates for MI patients were assumed to be the same as the US population because studies suggest that 2-year mortality rates after MI are comparable between the two populations [13, 14, 18]. Mortality rates for angina and heart failure patients were based on Ontario data [19]. Mortality rates for heart disease free patients were calculated as the difference between all-cause mortality and mortality from heart disease, basing on data from Statistics Canada’s Canadian Mortality Database, as available online from the CANSIM Table “Cause of Death 2000-2011” [20]. Heart disease incidence rates were calculated from the NPHS, which is a longitudinal health survey [21]. It started in 1994/1995 with an initial sample size of 17276 and ended in 2012 after another 8 follow-ups, which was conducted every two years.

### Statistical analysis

The incidence rate of the outcome of interest in each age- and sex- group is estimated by equation (1), where *I*_*i*_ is the incidence, *F*_*i*_ approximates the proportional cohort size change over the time interval between two surveys (which was given by equation (2)), *SP*_*i*_ is the proportion of disease-positive patients at the beginning of the first survey who survive to the next survey, *SN*_*i*_ is the corresponding proportion for the disease-negative people, *p*_*i,0*_ is the prevalence of outcome of interest at the first survey, *p*_*i,T*_ is the prevalence at the second survey respectively. In this paper, the time intervals for both the NHANES and the CCHS were 2 years. Equation (1) calculates the point estimates of incidence. Poisson-based confidence intervals for incidence can be estimated, treating the survey as a simple random design, without considering the survey design and sampling procedure. After obtaining the crude incidence rate for each age- and sex- group, a standardized incidence for the population is calculated using the direct method. For the United States cohort, we used the total US population in 2000 as the standard population; for the Canadian cohort, we used the total Canadian population from the 2001 Census. As each wave of the NHANES and the CCHS covers two years (e.g. 1999–2000, 2001–2002), the incidence estimated from such two waves was presented as between January 1st of the second year of each wave, i.e.,between Jan 1, 2000, and Jan 1, 2002.

Sex-specific five-year age group was used as the smallest analytic unit. Prevalence estimates for each age- and sex- group were calculated with proper sample weights and survey design effect used by the NHANES and the CCHS. It was substituted with the value from next age group if the prevalence was zero in the very young age groups. Baseline 2-year mortality after MI was calculated with equation (3), for both the US population and the Canadian population, where *M*_*MIin*_ denotes MI mortality in hospital, and *M*_*MIout*_ denotes MI mortality after hospital discharge. A 3% decrease in mortality rate for MI positive patients each year was assigned according to the literature [22]. The 2-year survival fraction for MI patients (*SP*_*MI*_) was given by equation (4). Baseline 2-year survival fraction after heart disease (*SP*_*HD*_) for the Canadian population was calculated with equation (5), where *w*_*MI*_, *w*_*AG*_, *w*_*HF*_ denotes the weighted proportion of MI/angina/heart failure to the sum of the three (in the CCHS cycle 2001). The mortality rate for MI or heart disease negative people were calculated with equation (6) and (7), as the difference between all-cause mortality (*MV*_*All* − *cause*_) and MI/heart disease mortality (*MV*_*MI*_, *MV*_*HD*_) from population vital statistics. The observed incidences of hospitalized MI in the US population were retrieved from CDC report, epidemiological studies and population statistics [2, 16, 23]. The observed incidences of heart disease in the Canadian population were calculated with corresponding weights from NPHS. In this paper, we will use the term “estimated” to refer to model calculated incidence, and the term “observed” to refer to incidence estimated from a cohort study, or reported in the literature or population statistics.

1 $$ {I}_i\approx \frac{2\left({F}_i{p}_{i,T}-S{P}_i{p}_{i,0}\right)}{T\left(1-{p}_{i,0}+{F}_i\left(1-{p}_{i,T}\right)\right)} $$

2 $$ {F}_i\approx 1-\left(1-S{P}_i\right){p}_{i,0}-\left(1-S{N}_i\right)\left(1-{p}_{i,0}\right) $$

3 $$ {M}_{MI}={M}_{MIin}+\left(1-{M}_{MIin}\right)\ast {M}_{MIout} $$

4 $$ S{P}_{MI}=1-{M}_{MI} $$

5 $$ S{P}_{HD}=1-{M}_{MI}\ast {W}_{MI}-{M}_{AG}\ast {W}_{AG}-{M}_{HF}\ast {W}_{HF} $$

6 $$ S{N}_{MI}=1-\left(M{V}_{All- cause}-M{V}_{MI}\right) $$

7 $$ S{N}_{HD}=1-\left(M{V}_{All- cause}-M{V}_{HD}\right) $$

To validate the model performance, we compared the estimated incidence with observed values. We checked whether our estimated incidence fall into the high-low range (or 95% CIs) of observed incidence. When the definition of best available reported outcome is different from what we modeled, we adjusted our estimated values to make a fair comparison. For example, the most reliable statistics in the US population was hospitalized MI incidence. Thus we adjusted downward our estimated values, considering about 30% of MI would be silent cases, and another 20% would be fatal cases, to be more comparable [4, 24, 25]. The time trend of estimated incidence was investigated by fitting a linear regression, with estimated incidence as the y outcome and survey year minus 2000 as the single x predictor.

## Results

### US Data

**Fig. 1:**
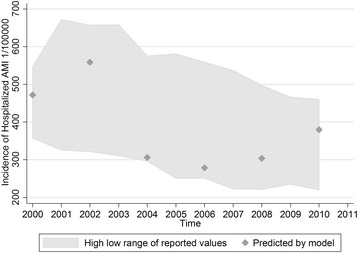
Observed and modelled hospitalized myocardial infarction incidence rate in the US from 2000 to 2011. The high/low boundary of the grey area represents the highest/lowest hospitalized myocardial infarction rate at state level recorded in the national environmental public health tracking network of U.S. Centers for Disease Control and Prevention. The diamond points represent the estimated national hospitalized myocardial infarction rate by our model

Estimated MI incidence in the US population (aged 35 years and older) from 2000 are provided in Table 1. The estimated MI incidence was 843 (1/100,000) between 2000 and 2001, and 678 (1/100,000) from 2010 to 2011, with a decreasing trend. The highest estimated MI incidence from the model was observed in 2002–2003. We also estimated hospitalized MI incidence to be 56% of MI incidence. Linear regression showed that the MI incidence decreased by approximately 32 (1/100,000) each year since 2000. The corresponding decrease in hospitalized MI was approximately 18 (1/100,000) respectively, which translated to a 3.8% decrease annually. We projected a 38% reduction in hospitalized MI incidence from 2000 to 2010, based on the prevalence from NHANES. Our hospitalized MI incidence estimates were compared against the reported values from those States which participated in the CDC environmental public health tracking program from 2000 to 2011 (Fig. 1). All of our estimated incidences fell into the high-low range of the reported values.

**Table 1:** Estimated incidence rate of all and hospitalized myocardial infarction in the US population from 2000 to 2011 (1/100000)

	2000–2001	2002–2003	2004–2005	2006–2007	2008–2009	2010–2011
Estimated incidence of all MI	843	998	546	498	543	678
Estimated incidence of hospitalized MI^a^	472	559	306	279	304	380

^a^56% of all the myocardial infarctions will be hospitalized basing on the assumption that 30% of the myocardial infarction events are fatal, and 20% of the myocardial infarction are silent (thus not hospitalized).

Incidence of myocardial infarction = 843-32*(year-2000); Incidence of hospitalized myocardial infarction = 540-18*(year-2000)

### Canadian data

**Fig. 2:**
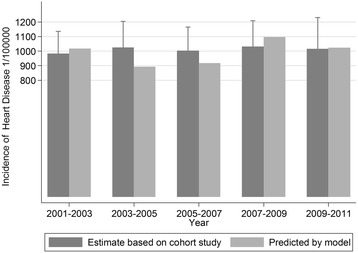
Observed and modelled incidence of heart disease in the Canadian population from 2001 to 2011. The *dark grey* bars with confidence intervals represent the estimated incidence of heart disease from the National Population Health Survey. The light grey bars represent the estimated incidence of heart disease from our model. All the predicted values were within the 95% CIs of the observed incidence. The average difference between the predicted values and the observed incidence were less than 10%

The model estimated heart disease incidence from CCHS, and observed heart disease incidence (with 95% CIs) from NPHS, for the Canadian population aged 12 years and older are shown in Table 2. We modelled two sets of heart disease incidence, with one set standardized to the NPHS age structure, and another set standardized to 2001 Canadian Census population. Our estimated heart disease incidence (first set) were very close to the NPHS values, with the smallest relative difference observed in 2009–2011 (0.7%), and the largest observed in 2003–2005 (12.9%). All of our first set estimated heart disease incidence fell into the 95% CIs of NPHS heart disease incidence. Figure 2 visually depicts how close between the NPHS incidence and our estimated incidence. The second set of heart disease incidence showed a decreasing trend among the Canadian population from 2001 to 2011. On average, heart disease incidence decreased by 13 (1/100,000) each year from 2001. The difference between the two sets of estimated heart disease incidence was due to the different age structures of the NPHS and the 2000 Canadian Census we used during direct standardization.

**Table 2:** Incidence rate of heart diseases in NPHS cohort and model estimates for the Canadian population from 2001 to 2011 (1/100000)

	2001–2003	2003–2005	2005–2007	2007–2009	2009–2011
NPHS incidence	983	1025	1003	1031	1016
lower 95% CI of reported incidence	831	845	840	852	802
upper95% CI of reported incidence	1136	1204	1165	1209	1230
Estimated incidence 1^a^	1018	893	918	1097	1023
Estimated incidence 2^b^	927	779	783	873	752

^a^Estimated incidence 1, standardized to NPHS age structure

^b^Estimated incidence 2, standardized to 2000 Canadian census population

Incidence of heart disease (standardized to 2000 Canadian census population) = 887-13*(year-2000)

We provide the model input, which is also part of the results, in the Appendix. The mortality rate of acute MI during hospitalization, the 2-year mortality rate of acute MI after hospital discharge, and the survival fraction 2 years after acute MI for the U.S. population in 1999 are presented in Table 3 in Appendix. The prevalence of MI in the U.S. population from 1999 to 2009 (in a 2-year time interval) estimated from NHANES are shown in Table 4 in Appendix. Table 5 in Appendix shows the 2-year mortality rate after MI, angina, heart failure, and the constructed 2-year survival fraction after heart disease. Table 6 in Appendix presents the prevalence of heart disease in the Canadian population, calculated from CCHS 2001 to 2011. Hospitalized MI incidence data from 26 States which participated in the CDC environmental public health tracking program (from 2000 to 2012) are provided in Table 7 in Appendix. Table 8 and 9 in Appendix provide the population age structure of the U.S. population and the Canadian population used for standardization, respectively.

## Discussion

A useful prevalence-to-incidence method was tested and validated for cardiovascular diseases in the general population of USA and Canada in this paper. Accurate estimates of cardiovascular disease incidence are still not available for populations not served by electronic health care information systems, or representative longitudinal surveys. Such data are lacking for sub- populations undergoing rapid health transition, with a rapidly increasing burden of cardiovascular diseases, such as indigenous people in North America. The prevalence-to-incidence method offers an alternative option to monitor, and compare the emerging cardiovascular disease pandemic, both globally and locally. Although we tested the method using cardiovascular health outcomes, it should also apply in other chronic conditions as long as the condition is irreversible

Estimates of hospitalized MI incidence in the U.S. population were in very close agreement with the actual statistics [16, 23]. Our MI incidence estimates can theoretically capture all MI cases. Hospitalized MI incidence miss those individuals who do not receive medical care, or died before reaching care. To better compare and validate our method, we made assumptions to convert all MI incidence to hospitalized MI incidence based on literature [4, 24, 25]. All of our estimated incidences fell into the high-low range of reported hospitalized MI incidence in the corresponding year. Estimates of hospitalized MI incidence were close to the low end of reported values in certain years. Several reasons might explain that. First, the NHANES was designed to represent the U.S. population demographically, its prevalence estimates for a certain disease varied across waves to some extent. For example, the prevalence of MI in female in the 2009 wave was substantially lower, while the prevalence in male in the 2003 wave was substantially higher than neighboring waves. The fluctuation in prevalence would lead to variation in incidence estimates. Second, the definitions of MI in the NHANES and Tracking Network of CDC might not match exactly. What NHANES recorded was the self-reported doctor confirmed MI. The self-reported data generally suffered from recall bias, compared to the hospital discharge data used by the CDC Tracking Network. Third, this study used data covering more than 10 years. Changes in diagnostic techniques and criteria, in the coding of MI, or in medical care access may all contribute to the fluctuation of MI incidence, both estimated from the model and reported by the CDC Tracking Network [26]. In addition, we used the same ratio to adjust overall MI incidence to hospitalized MI incidence for all the years, and that might also introduce some uncertainties.

A declining trend was observed from our hospitalized MI incidence estimates, as well as the reported values from the Tracking Network of CDC. Another study showed a very similar trend of acute MI incidence rate from 1999 and 2008 in Northern California, the incidence of which also peaked around the year 2001 and then decreased gradually [11]. Our method yielded an average annually 3.8% decrease of hospitalized MI incidence in the general U.S. population, comparing to a 2.4% decrease in acute MI in North California from 1999 to 2008 [11], a 5.8% decrease in acute MI among the Medicare fee-for-service beneficiaries from 2002 to 2007 [27], and a 4.9% decrease in age- and biomarker-adjusted incidence of hospitalization for AMI or fatal CHD in the Atherosclerosis Risk in Communities Study (ARIC) from 1987 to 2008 [28]. As summarized above, the decreasing rate identified from our estimates was also in accordance with reported values from cohort studies or surveillance data.

We further tested our method in the Canadian population. The NPHS and the CCHS share similar survey framework and the same questionnaire for chronic conditions [17, 21]. Our heart disease incidence estimates from different cycles of the CCHS were very close to the NPHS heart disease incidence when we standardized to the NPHS age structure. That result demonstrated that our method could provide accurate incidence estimate as a cohort study. More interestingly, the heart disease incidence estimates decreased constantly since 2001 if we standardized the results to the Canadian census population. This fact showed that our method was actually superior to closed cohort study (without buy-in participants during follow-up), in terms of estimating the incidence and its trend over time. It would be more consistent if we validated our method using MI as the outcome in the Canadian population first, however, unfortunately, the CCHS stopped to ask about MI since 2004. We derived the mortality of heart disease based on information for MI, angina and heart failure. The good agreement between incidence estimates and cohort study values demonstrated that other than a single health outcome (e.g. MI), this method could also be used for health outcomes with multiple components, e.g. heart disease (MI, angina, and heart failure), potentially stroke (ischemic, hemorrhagic, and unspecified) and other health outcomes.

Our modelling method also has limitations. It depends on the quality of prevalence and mortality data, and the extent of such data available. Assumptions and robust sensitivity analyses become essential in some circumstances. Our method was tested in short survey interval and small age- and sex- group basis. The combined incidences for male and female, and for all ages were compared against cohort study or population statistics. Future work is planned to test the model performance for subgroups, e.g. male and female separately, or in certain specific age group. The application of this method in other chronic conditions also needs to be tested, especially when their prevalence and mortality differ from MI or heart disease substantially.

## Conclusion

In conclusion, a reliable prevalence to incidence method for cardiovascular health outcomes was tested and validated. The incidence estimates given by the method were on average within 10% of the values from a cohort study. This method could also capture the trend of incidence if multiple cross-sectional data are available. This method has the potential to be used in population without valid cardiovascular disease incidence statistics.

## Appendix

This files includes 7 tables with the following data: 1) Cohort mortality for US population with and without acute myocardial infarction in 1999, 2) Prevalence of myocardial infarction in NHANES participants (%), 3) Cohort mortality for Canadian population with and without heart disease in 2001, 4) Prevalence of heart disease in CCHS participants (%), 5) Hospitalized MI incidence in different states in the U.S. from 2000 to 2012, 6) Age distribution by sex, 2000 US population (%), 7) Age distribution by sex, 2001 Canadian population (%).

**Table 3:** Cohort mortality for US population with and without acute myocardial infarction in 1999^a^

Age group	Mortality rate during hospitalization (a)	2-year mortality rate after hospital discharge (b)	Overall 2-year mortality rate after AMI^b^ (c)	Survival fraction 2 years after AMI^c^ (d)	1-year mortality for people without AMI (e)
Men					
35–49	0.029	0.060	0.087	0.913	0.003
50–54	0.041	0.083	0.121	0.879	0.006
55–59	0.057	0.083	0.135	0.865	0.009
60–64	0.082	0.172	0.240	0.760	0.014
65–69	0.107	0.172	0.261	0.739	0.022
70–74	0.144	0.289	0.391	0.609	0.034
75–79	0.184	0.289	0.420	0.580	0.052
80+	0.218	0.513	0.619	0.381	0.083
Women					
35–49	0.061	0.120	0.174	0.826	0.002
50–54	0.074	0.115	0.180	0.820	0.004
55–59	0.095	0.115	0.199	0.801	0.006
60–64	0.111	0.184	0.275	0.725	0.009
65–69	0.134	0.184	0.293	0.707	0.014
70–74	0.166	0.310	0.425	0.575	0.022
75–79	0.191	0.310	0.442	0.558	0.035
80+	0.215	0.460	0.576	0.424	0.057

^a^3% improve in overall 2-year AMI mortality in every two years were assumed for subsequent years

^b^c = a + (1-a)*b

^c^d = 1-c

**Table 4:** Prevalence of myocardial infarction in NHANES participants (%)^a^

	1999	2001	2003	2005	2007	2009	2011
Men							
35–39	0.19 (0.31)	1.11 (0.74)	0.33 (0.43)	0 (0)	0.42 (0.4)	0.41 (0.4)	0.45 (0.44)
40–44	2.72 (1.13)	2.41 (0.99)	1.32 (0.79)	0.19 (0.3)	0.61 (0.52)	2.03 (0.88)	1.66 (0.86)
45–49	4.78 (1.72)	0.24 (0.33)	1.78 (0.98)	4.48 (1.45)	2.67 (1.07)	0.25 (0.31)	1.17 (0.75)
50–54	3.37 (1.42)	3.81 (1.31)	5.49 (1.73)	1.81 (0.96)	1.99 (0.84)	5.84 (1.43)	2.46 (1.02)
55–59	15.21 (3.26)	8.77 (2.37)	9.00 (2.63)	4.57 (1.82)	5.91 (1.72)	8.68 (1.86)	4.48 (1.46)
60–64	15.16 (2.43)	8.25 (1.97)	9.55 (2.13)	10.4 (2.23)	7.47 (1.54)	8.81 (1.76)	7.16 (1.58)
65–69	8.78 (2.06)	14.76 (2.8)	18.25 (2.86)	15.42 (2.85)	14.61 (2.52)	10.29 (2.06)	11.33 (2.30)
70–74	14.72 (2.72)	10.20 (2.29)	19.45 (2.93)	11.20 (2.58)	13.23 (2.52)	14.22 (2.55)	16.70 (3.10)
75–79	16.46 (3.24)	18.81 (3.51)	11.82 (2.7)	21.68 (3.89)	16.75 (2.88)	16.89 (3.16)	10.38 (2.87)
80+	17.47 (2.78)	20.83 (2.67)	21.99 (2.8)	22.73 (3.06)	22.34 (2.98)	17.29 (2.67)	14.93 (2.77)
Women							
35–39	0 (0)	0.36 (0.38)	0 (0)	0 (0)	0.79 (0.55)	0.47 (0.41)	0 (0)
40–44	0.08 (0.2)	0.73 (0.55)	0.41 (0.44)	1.89 (0.91)	0.78 (0.56)	1.74 (0.75)	1.53 (0.79)
45–49	0.63 (0.57)	0.14 (0.25)	2.79 (1.2)	0.88 (0.66)	1.80 (0.84)	1.87 (0.8)	0.54 (0.49)
50–54	1.36 (0.86)	2.01 (1.02)	3.19 (1.29)	3.52 (1.37)	2.35 (0.98)	1.9 (0.85)	0.90 (0.58)
55–59	1.18 (0.99)	2.90 (1.41)	4.57 (1.85)	1.93 (1.18)	1.89 (0.94)	1.54 (0.88)	4.34 (1.42)
60–64	3.79 (1.28)	5.97 (1.63)	4.57 (1.43)	4.24 (1.46)	3.93 (1.12)	3.16 (1.03)	2.70 (0.98)
65–69	6.28 (1.78)	4.39 (1.58)	6.50 (1.83)	5.87 (1.95)	3.94 (1.37)	1.79 (0.96)	5.73 (1.76)
70–74	4.71 (1.65)	8.31 (2.18)	8.15 (2.21)	6.78 (2.18)	8.16 (1.91)	2.51 (1.07)	11.13 (2.53)
75–79	7.65 (2.39)	13.50 (3.03)	11.38 (2.81)	7.53 (2.78)	5.39 (1.80)	1.84 (1.14)	4.55 (2.05)
80+	9.92 (1.95)	11.44 (1.81)	12.63 (1.93)	11.29 (2.21)	12.45 (2.13)	8.54 (1.88)	11.20 (2.26)

^a^Estimated with proper weighting variables and survey design. Mean (SE) for all values

**Table 5:** Cohort mortality for Canadian population with and without heart disease in 2001^a^

Age group	2- year mortality rate after MI (a)	2-year mortality rate after angina (b)	2-year mortality rate after heart failure (c)	Survival fraction 2 years after IHD (d)	1-year mortality for people without IHD (e)
Men					
15–34	0.043	0.012	0.180	0.952	0.001
35–49	0.087	0.018	0.180	0.915	0.001
50–54	0.121	0.024	0.190	0.908	0.002
55–59	0.135	0.032	0.300	0.826	0.002
60–64	0.240	0.032	0.300	0.838	0.004
65-69	0.261	0.058	0.470	0.729	0.006
70–74	0.391	0.058	0.470	0.718	0.010
75–79	0.420	0.130	0.640	0.545	0.015
80+	0.619	0.130	0.640	0.553	0.025
Women					
15–34	0.086	0.014	0.220	0.957	0.001
35–49	0.174	0.120	0.174	0.924	0.001
50–54	0.180	0.020	0.110	0.858	0.001
55–59	0.199	0.028	0.320	0.829	0.002
60–64	0.275	0.028	0.320	0.775	0.003
65–69	0.293	0.050	0.430	0.756	0.004
70–74	0.425	0.050	0.430	0.743	0.006
75–79	0.442	0.108	0.560	0.649	0.010
80+	0.576	0.108	0.560	0.636	0.016

^a^d = 1- (weight_a_*a + weight_b_*b + weight_c_*c). weight_a_ = proportion of MI to all heart disease in CCHS 2001 wave; weight_b_ = proportion of angina to all heart disease in CCHS 2001 wave; weight_c_ = proportion of heart failure to all heart disease in CCHS 2001 wave. CCHS: Canadian Community Health Survey

**Table 6:** Prevalence of heart disease in CCHS participants (%)^a^

	2001	2003	2005	2007	2009	2011
Men						
15–19	0.47 (0.09)	0.59 (0.10)	0.62 (0.18)	0.52 (0.17)	0.52 (0.17)	0.18 (0.10)
20–24	0.58 (0.13)	0.90 (0.17)	0.55 (0.12)	0.83 (0.16)	0.83 (0.15)	1.00 (0.17)
25–29	0.57 (0.12)	0.52 (0.12)	0.48 (0.11)	0.55 (0.12)	0.55 (0.13)	0.66 (0.14)
30–34	0.75 (0.13)	0.71 (0.12)	0.79 (0.13)	0.42 (0.10)	0.42 (0.12)	0.58 (0.13)
35–39	1.12 (0.14)	1.14 (0.15)	1.03 (0.14)	0.80 (0.13)	0.80 (0.16)	0.98 (0.17)
40–44	1.90 (0.17)	2.02 (0.19)	1.89 (0.18)	1.76 (0.19)	1.76 (0.19)	1.26 (0.18)
45–49	3.23 (0.24)	3.48 (0.27)	3.19 (0.27)	2.79 (0.24)	2.79 (0.28)	3.24 (0.30)
50–54	6.23 (0.35)	5.87 (0.33)	5.55 (0.34)	5.74 (0.33)	5.74 (0.34)	5.27 (0.34)
55–59	10.59 (0.49)	9.49 (0.43)	9.91 (0.43)	8.80 (0.40)	8.80 (0.42)	9.19 (0.40)
60–64	12.99 (0.59)	14.10 (0.55)	12.50 (0.51)	12.34 (0.48)	12.34 (0.49)	12.94 (0.46)
65–69	18.36 (0.70)	17.78 (0.64)	17.51 (0.64)	17.87 (0.62)	17.87 (0.59)	16.02 (0.55)
70–74	22.54 (0.81)	21.84 (0.74)	22.27 (0.76)	21.31 (0.73)	21.31 (0.74)	20.45 (0.70)
75–79	27.47 (1.01)	26.53 (0.91)	25.96 (0.9)	24.22 (0.84)	24.22 (0.85)	22.78 (0.83)
80+	27.69 (1.04)	28.71 (0.96)	28.93 (0.93)	28.78 (0.87)	28.78 (0.86)	29.01 (0.81)
Women						
15–19	0.60 (0.10)	0.60 (0.10)	0.79 (0.20)	0.35 (0.14)	0.35 (0.20)	0.56 (0.19)
20–24	0.75 (0.14)	0.49 (0.12)	0.82 (0.14)	0.43 (0.11)	0.43 (0.10)	0.69 (0.14)
25–29	0.61 (0.11)	0.77 (0.13)	1.00 (0.14)	0.63 (0.12)	0.63 (0.12)	0.65 (0.12)
30–34	0.95 (0.13)	0.73 (0.12)	0.65 (0.11)	0.55 (0.10)	0.55 (0.13)	0.54 (0.11)
35–39	0.95 (0.12)	0.96 (0.13)	1.10 (0.15)	0.75 (0.12)	0.75 (0.12)	0.86 (0.14)
40–44	1.76 (0.16)	1.84 (0.19)	1.32 (0.15)	1.50 (0.17)	1.50 (0.16)	1.40 (0.18)
45–49	2.52 (0.21)	2.78 (0.22)	2.19 (0.21)	1.86 (0.19)	1.86 (0.24)	1.84 (0.22)
50–54	3.41 (0.25)	3.23 (0.23)	3.18 (0.23)	3.35 (0.23)	3.35 (0.26)	3.44 (0.25)
55–59	5.89 (0.36)	5.65 (0.30)	5.06 (0.29)	4.87 (0.27)	4.87 (0.27)	4.53 (0.26)
60–64	9.02 (0.46)	9.11 (0.42)	8.17 (0.39)	7.35 (0.34)	7.35 (0.33)	6.32 (0.30)
65–69	12.86 (0.55)	12.13 (0.49)	10.99 (0.48)	10.40 (0.45)	10.40 (0.42)	9.04 (0.38)
70–74	17.10 (0.62)	16.28 (0.56)	14.87 (0.56)	15.18 (0.56)	15.18 (0.54)	14.01 (0.53)
75–79	21.30 (0.72)	21.61 (0.67)	19.79 (0.67)	18.37 (0.63)	18.37 (0.62)	17.31 (0.62)
80+	26.19 (0.70)	26.34 (0.65)	24.91 (0.63)	24.08 (0.60)	24.08 (0.61)	23.41 (0.58)

^a^Estimated with proper weighting variables and survey design. Mean (SE) for all values

**Table 7:** Hospitalized MI incidence in different states in the U.S. from 2000 to 2012^a^

State	2000	2001	2002	2003	2004	2005	2006	2007	2008	2009	2010	2011	2012
Arizona	--	--	--	--	--	32.6	31.0	29.3	30.0	28.3	28.4	27.2	26.8
California	42.2	41.6	40.2	39.3	36.4	34.2	32.1	30.9	30.3	28.4	28.5	27.7	--
Colorado	--	--	--	--	30.5	28.4	27.8	25.5	24.6	23.5	22.0	21.3	21.0
Connecticut	46.5	43.1	42.4	41.8	38.9	35.3	31.7	31.6	33.5	30.6	29.2	26.6	29.1
Florida	50.2	49.2	47.9	46.6	43.7	41.3	38.0	36.4	35.5	33.0	32.8	30.6	30.9
Iowa	43.9	44.2	41.5	39.0	37.0	35.0	34.3	32.9	33.0	29.3	30.7	29.0	29.0
Kansas	42.3	39.6	38.3	38.2	36.3	34.0	33.1	30.3	30.2	28.2	26.5	26.6	26.3
Louisiana	37.1	38.9	40.9	37.5	37.2	30.2	29.9	30.1	31.3	32.3	31.7	33.3	--
Maine	--	67.1	65.6	65.6	57.3	57.8	55.4	53.2	49.7	46.6	46.0	45.2	--
Maryland	49.5	49.1	47.1	46.2	39.7	36.9	33.5	30.9	30.1	29.3	--	27.6	--
Massachusetts	50.6	51.0	50.9	51.1	45.4	42.1	40.1	37.8	35.9	34.0	32.8	30.8	30.1
Minnesota	42.7	42.3	40.6	37.8	35.8	35.9	33.8	31.3	31.0	28.8	27.8	26.7	26.9
Missouri	53.5	53.8	54.3	52.0	46.7	44.6	42.2	39.6	39.2	38.1	36.5	35.2	--
New Hampshire	50.2	48.6	45.4	45.7	40.7	38.7	37.8	38.4	36.0	33.7	27.9	--	--
New Jersey	50.1	48.8	49.2	46.7	42.9	39.9	37.9	36.3	37.0	35.4	35.2	33.7	33.6
New Mexico	35.7	35.0	32.9	31.8	30.5	27.3	31.7	30.0	28.9	27.1	26.2	--	--
New York	47.5	47.0	46.5	45.6	42.9	39.0	36.0	32.7	32.7	30.7	30.4	29.1	29.3
North Carolina	54.3	52.5	51.6	50.3	46.4	45.2	43.1	39.9	40.5	38.7	37.3	--	--
Oregon	35.0	34.7	35.7	34.5	31.5	30.2	29.9	29.6	28.8	26.7	24.9	24.6	24.7
Pennsylvania	56.5	54.4	53.4	52.0	48.1	44.8	42.7	40.1	39.7	37.4	35.8	35.1	35.0
South Carolina	50.5	49.4	47.8	45.7	41.5	40.5	39.1	37.4	36.4	33.4	32.9	32.6	31.8
Tennessee	53.1	52.1	53.6	51.1	45.8	45.5	45.2	43.1	42.4	41.1	42.4	41.4	--
Utah	36.6	33.7	33.8	30.7	27.6	24.8	25.1	22.1	22.5	21.1	21.1	22.6	21.4
Vermont	--	54.4	49.7	45.8	40.9	43.6	40.8	40.0	40.3	34.6	25.1	--	--
Washington	37.8	36.6	37.1	34.5	32.6	31.0	29.3	28.5	27.4	25.9	25.8	24.9	25.6
Wisconsin	43.6	43.5	41.8	39.1	37.4	35.6	33.5	32.2	31.7	29.0	29.4	28.7	28.7

^a^Age standardized MI hospitalization rate for people aged 35 years and older

--no data available

**Table 8:** Age distribution by sex, 2000 US population (%)^a^

Age group	Male	Female
0–4	3.49	3.33
5–9	3.74	3.56
10–14	3.74	3.56
15–19	3.69	3.49
20–24	3.44	3.3
25–29	3.48	3.41
30–34	3.67	3.62
35–39	4.02	4.05
40–44	3.95	4.02
45–49	3.51	3.63
50–54	3.06	3.19
55–59	2.31	2.47
60–64	1.83	2.01
65–69	1.56	1.82
70–74	1.39	1.76
75–79	1.08	1.55
80–84	0.65	1.11
85+	0.44	1.07
Total Population	49.06	50.94

^a^Source: U.S. Census Bureau, Census 2000 and Census 2010. Web: www.census.gov

**Table 9:** Age distribution by sex, 2001 Canadian population (%)^a^

Age group	Male	Female
0–4	2.89	2.76
5–9	3.37	3.21
10–14	3.50	3.34
15–19	3.51	3.34
20–24	3.27	3.24
25–29	3.12	3.21
30–34	3.44	3.55
35–39	4.15	4.26
40–44	4.24	4.36
45–49	3.84	3.94
50–54	3.44	3.51
55–59	2.63	2.68
60–64	2.07	2.17
65–69	1.81	1.97
70–74	1.54	1.82
75–79	1.13	1.58
80–84	0.64	1.08
85+	0.42	0.97
Total Population	49.01	50.99

^a^Source: Statistics Canada - 2001 Census. Catalogue Number 95F0300XCB2001009

## Abbreviations

CCHS: Canadian Community Health Survey
CDC Tracking Network: The national environmental public health tracking network of U.S. Centers for Disease Control and Prevention
HD: Heart disease
MI: Myocardial infarction
NHANES: National Health and Nutrition Examination Survey
NPHS: National Population Health Survey
